# Analyses of medical coping styles and related factors among female patients undergoing in vitro fertilization and embryonic transfer

**DOI:** 10.1371/journal.pone.0231033

**Published:** 2020-04-03

**Authors:** Liwen Shen, Lanfeng Xing

**Affiliations:** 1 Zhejiang University School of Medicine, Hangzhou, Zhejiang, People's Republic of China; 2 Department of Center for Reproductive Medicine, Huzhou Maternity & Child Health Care Hospital, Huzhou, Zhejiang, People's Republic of China; 3 Department of Reproductive Endocrinology, Branch of National Clinical Research Center for Obstetrics & Gynecology,Women's Hospital, Zhejiang University School of Medicine, Hangzhou, Zhejiang, People's Republic of China; Peking University Third Hospital, CHINA

## Abstract

**Objective:**

This study investigated the medical coping styles of female patients treated with *in vitro* fertilization and embryonic transfer (IVF-ET), and analyzed the effects of alexithymia and social support on their choice of coping style.

**Methods:**

A survey was conducted with 285 female patients undergoing IVF-ET in a reproductive medical center of a third-grade class-A hospital in China using the Medical Coping Modes Questionnaire, the Social Support Rating Scale, and the Toronto Alexithymia scale.

**Results:**

Patients who underwent IVF-ET treatment had a higher score for avoidance as a coping mode than did normal controls. Utilization of social support predicted the use of confrontation as a coping style. Difficulty identifying feelings, objective support, and utilization of social support were factors in the choice of avoidance as a coping style, and length of infertility treatment, difficulty identifying feelings, and subjective support predicted patients’ use of the acceptance-resignation as a coping style.

**Conclusion:**

Patients who undergo IVF-ET generally select the coping style of avoidance, which is not conducive to treatment. Targeted intervention strategies should be developed based on the factors influencing patients’ choice of coping style(s) to guide them in choosing positive coping methods, improve compliance, and achieve successful pregnancy outcomes.

## Introduction

*In vitro* fertilization and embryonic transfer (IVF-ET) technology, one of the most significant scientific advances of the 20th century, is a major breakthrough in the field of assisted reproductive technology and a sign of hope for fertility among infertile women. However, due to the invasive nature of the procedure and uncertain outcome, patients can experience severe psychological stress [1]. Studies have shown that [2] the level of psychological stress is related to pregnancy outcomes of the infertility treatment, and that excessive stress is not conducive to pregnancy. Coping involves a variety of strategies and methods for dealing with threatening stressors and for alleviating or eliminating stress [3]. The choice of coping strategy determines whether patients in treatment of infertility adopt a positive or negative attitude to face infertility and comply with treatment, which ultimately affects the future course of treatment and even the success of the IVF-ET treatment [4, 5]. Although several studies have found a correlation between medical coping style and multiple psychological and emotional characteristics in female patients treated with IVF-ET [6], there are few studies on the factors influencing the choice of medical coping styles.

Social support has been reported to improve a variety of negative emotions and mental states, and help individuals with chronic conditions adapt psychologically and behaviorally [7]. Multiple studies have shown that depression and anxiety are common among infertile women [8]. Depression and anxiety are often accompanied by alexithymia [9], which is difficult to distinguish from personality traits or emotions. This study aimed to examine the medical coping style of patients undergoing IVF-ET treatment and analyze the influence of alexithymia and social support on their selection of medical coping styles to provide evidence-based recommendations for how to help patients adopt effective strategies to cope with medical issues and to improve their compliance with treatment.

## Materials and methods

### Ethics statement

The Ethics Committee of the obstetrics and gynecology hospital affiliated with Zhejiang University approved this study(Ethics approval number:20180067). All the study participants signed the informed forms before filling in the questionnaire, which ensured that the respondents voluntarily participated in the survey with full knowledge. All the questionnaires were completed in anonymously.

### Participants

This cross-sectional study was conducted with female patients who were treated with IVF-ET in the reproductive endocrinology clinic of a hospital. Data were collected from June 2018 to August 2018.The criteria for inclusion were as follows: age 20–40 years old; junior high school or higher educational level; the ability to read and answer questions independently; and voluntary participation in the survey. The exclusion criteria were as follows: experienced a major life event in the past 6 months; diagnosed with a severe physical or mental disorder; history of receiving donor sperm for IVF; history of a pre-implantation genetic diagnosis or pre-implantation genetic screening.

### Survey instruments

The researchers designed a general questionnaire after consulting the relevant literature. It consists of 13 items to collected data on respondents’ age, educational level, family’s annual income, length of marriage, cause of infertility, and birth history (number of children) of the infertile patients.

The Medical Coping Modes Questionnaire, developed by Feifel et al., [10] is one of the few scales designed to assess coping styles of patients’ who are experiencing medical events.Qianjin Jiang [11] adapted and standardized the Chinese version with consideration of China's cultural characteristics. The scale consists of 20 items that measure three dimensions (coping modes). Each item is scored on a 4-point scale ranging from 1 to 4. The three dimensions are confrontation, avoidance, and acceptance-resignation. A higher the cumulative score on each dimension indicates a greater likelihood that the respondent will use the corresponding coping mode.

The Toronto Alexithymia Scale was developed by Taylor et al^.^[12], and adapted for use with Chinese samples by Yonggui Yuan [13]. The scale is divided into three dimensions: difficulty identifying feelings (DIF), difficulty describing feelings (DDF), and externally oriented thinking (EOT). The scale yields dimension scores and a total score.

The Social Support Rating Scale was developed in China by Shuiyuan Xiao [14], and is widely used in studies on social support conducted in China. The scale has 10 items that measure three dimensions: objective support, subjective support, and utilization of support. The ratings of the items are summed for a total score of social support, with a higher score indicating better social support.

### Study procedures

Before the investigation was officially launched, training was conducted for the investigators who distributed the questionnaires, which contained an explanation of the study’s objectives. The patients signed the informed consent form at the same time the questionnaire was distributed to ensure their participation was voluntary and anonymous, and that their consent was given with their full knowledge of the study. Patients returned their questionnaires immediately after they completed them.

### Statistical analysis

The data were analyzed using SPSS 22 (IBM Corp., Armonk, NY). The descriptive data collected in the general questionnaire are summarized as frequencies and percentages. The total score for each scale and the scores on all of their dimensions are expressed as mean ± standard deviation. General demographic differences in medical coping modes were analyzed using the independent- samples t-test or one-way analysis of variance (ANOVA). Pearson’s correlations were used to analyze the relationships between alexithymia, social support, and medical coping modes. General demographic data, alexithymia, and the impact of social support on medical coping modes were analyzed using linear regression.

## Results

In this study, 320 questionnaires were distributed, 304 were returned and 19 invalid questionnaires were eliminated; 285 valid questionnaires were analyzed, with a return rate of 89.06%. Table 1 presents participants’ demographic data, clinical history, and treatment for infertility.

**Table 1:** Participants’ demographic and clinical characteristics.

Variable	Group	Number (persons)	Percentage %
Age (years)	20~34	213	74.7
	35~39	72	25.3
Residence	City	137	48.1
	Town	78	27.4
	Rural area	20	24.6
Living arrangement	Live only with husband	167	58.6
	Live with husband and in-laws	87	30.5
	Live with husband and parents	22	7.7
	Live with husband, in-laws, and husband’s brothers, and their children, and/or others.	9	3.2
Educational level	Junior high school, high school, or secondary school	125	43.9
	College/Bachelor’s degree	150	52.6
	Graduate and above	10	3.5
Employment	None	53	18.6
	Worker	11	3.9
	Farmer	4	1.4
	Office clerk	94	33
	Government official	9	3.2
	Teacher	21	7.4
	Medical personnel	16	5.6
	Self-employed	42	14.7
	Others	35	12.3
Annual household income (ten thousand yuan)	<3	25	8.8
	3~8	84	29.5
	9~14	100	35.1
	15~80	72	25.3
	>80	4	1.4
Length of the marriage (years)	<5	163	57.2
	5~10	106	37.2
	>10	16	5.6
Length of infertility (years)	<2	51	17.9
	2~4	174	61.1
	5~10	55	19.3
	>10	5	1.8
Birth history (number of children)	None	237	83.2
	One child		
	Two children and more	44	15.4
History of abortion (number)	None	157	55.1
	1	84	29.5
	2	30	10.5
	≥3	14	4.9
Cause of infertility	Female factor	184	64.6
	Male factor	23	8.1
	Both factors	37	13
	Unknown reason	41	14.4
Length of infertility treatment (years)	<1	108	37.9
	1~3 years	139	48.8
	>3	38	13.3
Infertility type	Primary infertility	149	52.3
	Secondary infertility	136	47.7

### Medical coping modes of patients treated with IVF-ET and normal controls

The use of avoidance as a coping mode was significantly higher among the patients treated with IVF-ET than the normal controls [11]. The use of acceptance-resignation was lower in the patients than the normal controls, but the difference was marginal, as shown in Table 2.

**Table 2:** Comparisons of scores on the medical coping modes questionnaire of patients treated with IVF-ET and normal controls.

Items	IVF-ET	Normal	t	P
**Confrontation**	19.22±3.00	19.48±3.81	-1.435	0.153
**Avoidance**	15.72±2.45	14.44±2.97	8.80	<0.001
**Acceptance-resignation**	8.54±2.38	8.81±3.17	-1.89	0.06

IVF-ET, in vitro fertilization and embryonic transfer.

### Medical coping modes and characteristics of patients treated with IVF-ET

The univariate analysis of the medical coping modes and demographic and clinical characteristics of patients treated with IVF-ET showed that length of the marriage, birth history, length of infertility treatment, and type of infertility of the patients who used acceptance-resignation as a coping mode were significantly different from that of other patients (Table 3).

**Table 3:** Medical coping modes and demographic and clinical characteristics of patients treated with IVF-ET.

Items	Groups	Confrontation	Avoidance	Acceptance-resignation
Age (years)	20~34	19.20±3.06	15.74±2.36	8.66±2.30
	35~39	19.31±2.85	15.64±2.71	8.19±2.59
	F/t value	0.07	0.095	2.079
	P-value	0.792	0.758	0.15
Residence	City	19.45±2.79	15.77±2.31	8.70±2.60
	Town	19.09±2.94	15.53±2.47	8.26±1.90
	Rural area	18.93±3.46	15.83±2.70	8.56±2.43
	F/t value	7.34	0.337	0.865
	P-value	0.445	0.714	0.422
Living arrangement	Live only with husband	19.32±2.96	15.89±2.44	8.80±2.48
	Live with husband and in-laws	19.39±3.11	15.59±2.48	8.21±2.32
	Live with husband and parents	19.00±2.4	15.23±2.27	7.77±1.5
	Live with husband, in-laws and husband’s brothers, and their children, and/or others	16.44±3.13	14.89±2.76	9.00±2.35
	F/t value	2.804	1.01	2.107
	P-value	0.4	0.391	0.099
Educational level	Junior high school, high school, or secondary school	18.81±3.45	15.54±2.41	8.30±2.42
	College/Bachelor’s degree	19.59±2.61	15.81±2.42	8.64±2.27
	Graduate and above	19.00±1.94	16.40±3.34	10.10±3.14
	F/t value	2.341	0.816	2.927
	P-value	0.098	0.443	0.055
Employment	None	18.94±3.16	15.57±2.41	9.02±2.51
	Worker	19.73±3.74	16.36±3.14	8.181.89
	Farmer	18.25±5.06	16.25±2.36	7.25±0.96
	Office clerk	19.27±2.85	15.62±2.68	8.56±2.71
	Government official	18.78±2.22	15.67±2.78	7.67±1.73
	Teacher	19.48±2.64	16.14±2.10	9.05±1.88
	Medical personnel	20.25±2.05	15.81±2.17	9.44±2.03
	Self-employed	19.64±3.32	15.98±2.25	7.95±2.15
	Others	18.49±3.07	15.34±2.24	8.26±2.12
	F/t value	0.789	0.402	1.396
	P-value	0.613	0.919	0.198
Annual household income (ten thousand yuan)	<3	19.68±4.09	16.04±2.48	9.64±2.96
	3~8	18.56±3.33	15.25±2.33	8.14±2.10
	9~14	19.54±2.63	15.86±2.50	8.71±2.44
	15~80	19.36±2.61	15.99±2.52	8.43±2.32
	>80	20.00±2.449	15.00±1.41	8.00±2.45
	F/t value	1.564	1.266	2.17
	P-value	0.184	0.283	0.073
Length of marriage (years)	<5	19.06±3.07	15.76±2.38	8.40±2.15
	5~10	19.45±2.88	15.65±2.48	8.95±2.73
	>10	19.38±3.18	15.69±3.01	7.31±1.66
	F/t value	0.565	0.065	4.09
	P-value	0.569	0.937	0.018
Length of infertility (years)	<2	18.76±3.03	15.47±2.41	8.31±2.16
	2~4	19.39±3.08	15.92±2.46	8.39±2.25
	5~10	19.09±2.76	15.42±2.28	9.11±2.84
	>10	19.8±2.78	14.4±3.78	10.00±3.00
	F/t value	0.659	1.329	2.075
	P-value	0.578	0.265	0.104
Birth history (children)	None	19.13±2.84	15.69±2.33	8.74±2.40
	One child	19.73±3.75	15.80±3.06	7.57±2.11
	Two children and more	19.25±3.59	16.25±2.63	7.50±1.29
	F/t value	0.73	0.129	5.035
	P-value	0.483	0.879	0.007
History of abortion (number)	None	19.17±2.95	15.71±2.32	8.64±2.51
	1	19.44±2.93	15.63±2.48	8.60±2.30
	2	18.63±3.34	15.43±2.27	8.23±2.00
	≥3	19.86±3.44	16.93±3.71	7.86±2.14
	F/t value	0.757	1.317	0.648
	P-value	0.519	0.269	0.585
Cause of infertility	Female factor	19.10±2.97	15.72±2.52	8.48±2.37
	Male factor	18.96±2.65	14.91±2.11	8.83±1.88
	Both factors	19.32±2.77	15.70±2.22	8.84±288
	Unknown reason	19.83±3.54	16.17±2.44	8.39±2.26
	F/t value	0.726	1.301	0.389
	P-value	0.537	0.274	0.761
Length of infertility treatment (years)	<1	19.05±2.92	15.87±2.47	7.92±2.03
	1~3 years	19.17±3.06	15.72±2.46	8.77±2.50
	>3	19.95±3.00	15.26±2.37	9.50±2.45
	F/t value	1.32	0.864	7.779
	P-value	0.269	0.423	0.001
Infertility type	Primary infertility	19.19±2.91	15.81±2.44	8.88±2.51
	Secondary infertility	19.26±3.12	15.61±2.47	8.18±2.19
	F/t value	0.031	0.482	6.299
	P-value	0.861	0.488	0.013

### Correlations of medical coping modes with alexithymia among patients treated with IVF-ET

Table 4 shows the correlations of medical coping modes with the alexithymia total and dimension scores of the patients treated with IVF-ET. The alexithymia total score had a significant positive correlation with acceptance-resignation, DIF had a significant positive correlation with avoidance and acceptance-resignation, and EOT had a significant negative correlation with confrontation.

**Table 4:** Correlation analysis of alexithymia and medical coping modes among patients treated with IVF-ET (r).

Dimension	Confrontation	Avoidance	Acceptance-resignation
**Alexithymia**	-.43	.079	.169**
**DIF**	.026	.143*	.203**
**DDF**	-.045	.045	.100
**EOT**	-.121*	-.043	.069

DIF, difficulty identifying feelings; DDF, difficulty describing feelings; EOT, externally oriented thinking

*P < 0.05

** P < 0.01.

Table 5 shows the analysis of the correlations between social support and the coping modes of the patients treated with IVF-ET. Total social support and all three dimensions of social support had significant negative correlations with acceptance-resignation and significant positive correlations with avoidance. All measures except objective support also had positive significant positive correlations with confrontation.

**Table 5:** Correlation analysis of social support and medical coping modes in female patients treated with IVF-ET (r).

Dimension	Confrontation	Avoidance	Acceptance-resignation
**Social support**	.207**	.212**	-.269**
**Objective support**	.110	.180**	-.195**
**Subjective support**	.151*	.123*	-.234**
**Utilization of support**	.264**	.240**	-.161**

* P < 0.05

** P < 0.01.

### Multivariate regression of medical coping modes on the demographic and clinical factors

The statistically significant variables identified in the univariate analysis (length of marriage, birth history, length of infertility treatment, and infertility type), were the independent variables in the regression analysis and acceptance-resignation was the dependent variable (Table 6). The results showed that length of infertility treatment significantly predicted the use of acceptance-resignation as a coping mode by the patients treated with IVF-ET.

**Table 6:** Multivariate analysis of the acceptance-resignation coping mode among the patients treated with IVF-ET.

**Acceptance- resignation coping mode**	**Variable**	**Regression coefficient**	**Standard regression coefficient**	**t**	**P**
	Marriage length	0.181	0.046	0.713	0.476
	Birth history	-0.703	-0.124	0.053	0.053
	Length of infertility treatment	0.704	0.199	3.33	0.001
	Infertility type	-0.532	-0.122	-1.75	0.081

R = 0.291, R^2^ = 0.085, adjusted R^2^ = 0.072, F = 6.478.

### Multiple regression of medical coping modes on alexithymia and social support

The statistically significant variables identified in the correlation analysis (EOT, subjective support, and utilization of support) were the independent variables, and confrontation was the dependent variable for the regression analysis. Utilization of support significantly predicted the use of confrontation as a coping mode by patients treated with IVF-ET, as shown in Table 7.

**Table 7:** Multivariate analysis of the confrontation coping mode among the patients treated with IVF-ET.

**Confrontation coping mode**	**Variable**		**Regression coefficient**		**Standard regression coefficient**		**t**		**P**
	(Constant)		17.369				9.885		<0.001
	EOT	-0.090		-0.084		-1.440		0.151	
	Subjective support	0.040		0.062		0.997		0.320	
	Utilization of support	0.383		0.227		3.620		<0.001	

EOT, externally oriented thinking; R = 0.282, R^2^ = 0.079, adjusted R^2^ = 0.069, F = 8.069.

The statistically significant variables from the correlation analysis (DIF, objective support, subjective support, and utilization of support) were the independent variables and avoidance was the dependent variable for the multiple regression analysis. The analysis showed that DIF, objective support, and the utilization of support significantly predicted the use of the avoidance as a coping mode by the patients treated with IVF-ET (Table 8).

**Table 8:** Multivariate analysis of the avoidance coping mode among patients treated with IVF-ET.

**Avoidance coping mode**	**Variable**	**Regression coefficient**	**Standard regression coefficient**	**t**	**P**
	(Constant)	10.218		9.556	<0.001
	DIF	0.113	0.203	3.494	0.001
	Subjective support	-0.003	-0.005	-0.086	0.931
	Objective support	0.128	0.152	2.413	0.016
	Utilization of support	0.296	0.215	3.394	0.001

DIF, difficulty identifying feelings; R = 0.328, R^2^ = 0.109, adjusted R^2^ = 0.095, F = 2.329.

The statistically significant variables from the correlation analysis (total scores for alexithymia, DIF, social support, objective support, subjective support, and utilization of support) were the independent variables and acceptance-resignation was the dependent variable for the regression analysis. The results showed that DIF and subjective support significantly predicted the use of acceptance-resignation as a coping mode, as shown in Table 9.

**Table 9:** Multivariate analysis of the acceptance-resignation coping mode among patients treated with IVF-ET.

**Acceptance-resignation coping mode**	**Variable**	**Regression coefficient**	**Standard regression coefficient**	**t**	**P**
	(Constant)	9.903		9.490	<0.001
	DIF	0.096	0.178	3.051	0.002
	Subjective support	-0.098	-0.191	-3.030	0.003
	Objective support	-0.066	-0.081	-1.281	0.201
	Utilization of support	-0.050	-0.038	-0.592	0.554

DIF, difficulty identifying feelings; R = 0.320, R^2^ = 0.090, adjusted R^2^ = 0.088, F = 8.002.

## Discussion

This study investigated the medical coping styles of female patients treated with IVF-ET using a questionnaire survey, and found that their use of avoidance as a medical coping mode was significantly higher than that of normal controls, which is similar to the results of other studies [15, 16]. Although the participants selected for these studies were at different stages of treatment, none of them used confrontation as a coping mode during their treatment for infertility. Avoidance and acceptance with resignation are often viewed as negative coping modes [17]; however, studies have suggested that [18] avoidance can be useful as a form of self-protection, which can reduce the likelihood of adverse reactions caused by stressful events over a short time. Therefore, female patients treated with IVF-ET might temporarily reduce negative psychological stress during their treatment for infertility by deliberately ignoring their negative moods and emotions as a way of diverting their attention. At the same time, patients who received IVF-ET treatment often undergo lengthy medical consultations and various examinations and procedures in the early stage of treatment, and their initial eagerness to have a child may have slowly decreased. Exorbitant costs, frequent round-trips to and from hospitals, taking various drugs orally and by injection, and fluctuations in hormone levels caused by the drugs can lead to physiological changes, and the accumulated pressure can become unbearable, forcing the patient to accept the reality of infertility.

Using confrontation as a coping method is a sign that a patient is taking a positive approach to disease [19]. Shi Xiao [20] found that improving social support could reduce patients' anxiety and depression, and increase the use of positive coping strategies. As suggested in this study, patients may actively seek help from others, use the support provided by others, and alleviate economic pressures, physiological adverse reactions, and negative emotions generated during IVF-ET treatment by envisaging the current diagnosis and treatment plan, and actively cooperating with the treatment.

Di Tella et al. [21] believe that in order to reduce the negative effects of adverse events, patients with a high level of DIF often adopt unusual coping strategies. Tominaga [22] has proposed that patients with high DIF scores on the Toronto Alexithymia Scale tend to use avoidance strategies. This study reached the same conclusion; DIF predicted patients’ use of avoidance as a coping mode. The patients who received IVF treatment may not have recognized or distinguished between physical and emotional feelings, and simply ignored their inner thoughts deliberately, perhaps because they realized it was impossible for them to identify the source of their feelings with accuracy, and it was difficult for others to understand their needs. At the same time, if the patients treated with IVF-ET were eager to seek help from others and had sufficient support and help from outsiders, they could have diverted their attention through the support and help from others, and talk about their inner thoughts.

The longer the infertility treatment lasts, the greater the patient’s tendency to use acceptance-resignation as a coping mode. IVF-ET treatment is the last resort for infertility treatment and most of the patients underwent ovulation induction, artificial insemination, and other procedures in the early stage, expending a considerable amount of energy and financial resources to complete the IVF-ET step. The initial eagerness to seek treatment actively may have been gone, and patients were more likely to consider the idea of accepting infertility as a reality. Smimi [23] believes that DIF is inversely related to general cognition, memory, and executive ability. This study also suggests that patients with difficulty recognizing emotions often adopted the ineffective attitude of acceptance-resignation when responding to IVF treatment. In contrast, when patients felt they had a strong supportive base and there were many people who cared about them and were willing to help, they were not likely to consider the idea of giving up treatment; instead, they believed that they would definitely conceive and give birth through hard work.

## Conclusion

This study is the first to investigate alexithymia and social support as factors influencing the choice of medical coping modes by patients undergoing IVF-ET. The results provide new insights for helping these patients improve their compliance during treatment. Based on this study’s results, the research team will actively explore effective psychological interventions, encourage patients to adopt effective coping methods, regain confidence, actively cooperate with treatment, and achieve satisfactory pregnancy outcomes.

A limitation of this study is that the research was conducted only in the largest maternal and child healthcare hospital in Zhejiang Province, and the people who diagnose and treat infertility are mainly in Zhejiang and the surrounding area, so the conclusion of this study is only applicable to the IVF treatment of women in Zhejiang Province.

## Supporting information

S1 Table The original data from questionnaires. (XLSX)
